# Biomimetic Peripheral Nerve Stimulation Promotes the Rat Hindlimb Motion Modulation in Stepping: An Experimental Analysis

**DOI:** 10.34133/cbsystems.0131

**Published:** 2024-07-04

**Authors:** Pengcheng Xi, Qingyu Yao, Yafei Liu, Jiping He, Rongyu Tang, Yiran Lang

**Affiliations:** ^1^ School of Mechatronical Engineering, Beijing Institute of Technology, Beijing, People’s Republic of China.; ^2^ National Engineering Research Center of Neuromodulation, Tsinghua University, Beijing, People’s Republic of China.; ^3^ Beijing Innovation Center for Intelligent Robots and Systems, Beijing Institute of Technology, Beijing, People’s Republic of China.; ^4^ Institute of Semiconductors, Chinese Academy of Science, Beijing, People’s Republic of China.; ^5^ School of Life Science, Beijing Institute of Technology, Beijing, People’s Republic of China

## Abstract

Peripheral nerve stimulation is an effective neuromodulation method in patients with lower extremity movement disorders caused by stroke, spinal cord injury, or other diseases. However, most current studies on rehabilitation using sciatic nerve stimulation focus solely on ankle motor regulation through stimulation of common peroneal and tibial nerves. Using the electrical nerve stimulation method, we here achieved muscle control via different sciatic nerve branches to facilitate the regulation of lower limb movements during stepping and standing. A map of relationships between muscles and nerve segments was established to artificially activate specific nerve fibers with the biomimetic stimulation waveform. Then, characteristic curves depicting the relationship between neural electrical stimulation intensity and joint control were established. Finally, by testing the selected stimulation parameters in anesthetized rats, we confirmed that single-cathode extraneural electrical stimulation could activate combined movements to promote lower limb movements. Thus, this method is effective and reliable for use in treatment for improving and rehabilitating lower limb motor dysfunction.

## Introduction

Disease- or injury-induced lower limb motor dysfunctions severely affect people’s quality of life. These dysfunctions can be triggered by various injuries and diseases such as spinal cord injury and stroke. Establishing a method for resolving such dysfunctions through electrical nerve stimulation has become a considerably researched topic. Currently, the available treatments in motion modulation include epidural electrical stimulation (EES) [1–4], surface neuromuscular electrical stimulation (NMES) [5–7], peripheral nerve stimulation (PNS) [8–14], transcranial direct current stimulation [15], and magnetic stimulation of spinal cord [16]. Compared with PNS, EES is more invasive and more likely to damage the central nervous system, while large differences exist in the available target locations of EES, which makes achieving mapping in this treatment difficult [17–19]. Further, NMES involves use of high-intensity stimulation during treatment and is therefore more likely to trigger muscle fatigue, which reduces the effectiveness of this approach [20]. The most vital factor, though, is that NMES involves nonlinear recruitment of muscle fibers [21], which makes controlling the stimulation parameters difficult. Therefore, in motor dysfunction treatment, PNS offers advantages, such as minimal invasiveness, simple means of achieving functional partition, and linearity of stimulation [22].

Sciatic nerve electrical stimulation [23] and femoral nerve electrical stimulation [24] are key electrical PNS methods for treating lower extremity motor dysfunction [8,25–27]. Sciatic nerve electrical stimulation was first performed in 1967 to address motor issues during stepping in patients with foot drop [28]. In the past 60 years, sciatic nerve electrical stimulation therapy has been primarily used for regulating the motor function in patients with foot drop. As electrode technology advanced, sciatic nerve electrodes have transformed from the single-channel format to the multichannel format [8,11,29], and stimulation targets can range from outside to inside [13,30–32].

In the stimulation paradigm of multicontact cuff electrodes, Veraart’s team [33] was the first to use a multicurrent source stimulation approach. The team demonstrated that muscle selectivity of multipoint stimulation is superior to that of single-point stimulation. Subsequently, Brill and colleagues [34,35], Guiraud and colleagues [36–39] widely adopted complex multicontact cuff electrodes and complex multicurrent sources on several contacts at the same time to achieve precise control on lower leg muscle groups. However, the method generally focuses on lower extremity motor regulation, specifically of the ankle. In fact, sciatic nerve splits into not only 2 main branches—the tibial nerve and common peroneal nerve—but also some small branches that innervate several muscles of the thigh on its course through the posterior thigh. Here, we proposed the electrical nerve stimulation at the proximal sciatic nerve segment to achieve effective control of multiple joint positions in a more concentrated stimulation position.

We here present a sciatic nerve stimulation method that will aid in lower extremity standing and stepping. First, the sciatic nerve was directionally stimulated using multichannel extrafascicular electrodes to establish the relationship between the stimulation site and the muscle. Then, using the joint motion data and evoked electromyography (EMG) signals, a sigmoid function relationship between the stimulation intensity and muscle activity was established. Finally, anesthetized rats were placed on a treadmill and stimulated to verify the selected stimulation parameters. Our results revealed that sciatic nerve electrical stimulation can help to effectively regulate the calf–ankle as well as standing and stepping movements during the swing/stance phase. Therefore, this method enables the accurate and effective regulation of lower limb motor muscles and offers a new experimental protocol for the rehabilitative treatment of patients with lower extremity dysfunctions.

## Materials and Methods

### Animals and implants

Six female Sprague–Dawley rats (age, 4 to 8 weeks) were used in the experiment. Each rat was reared in a separate cage at room temperature (25° ± 2 °C) and a humidity of 60%. Food and water were controlled to maintain the rats’ weights at 250 to 300 g. The animal experiments were approved by the Institutional Animal Care and Use Committee.

**Fig. 1.**
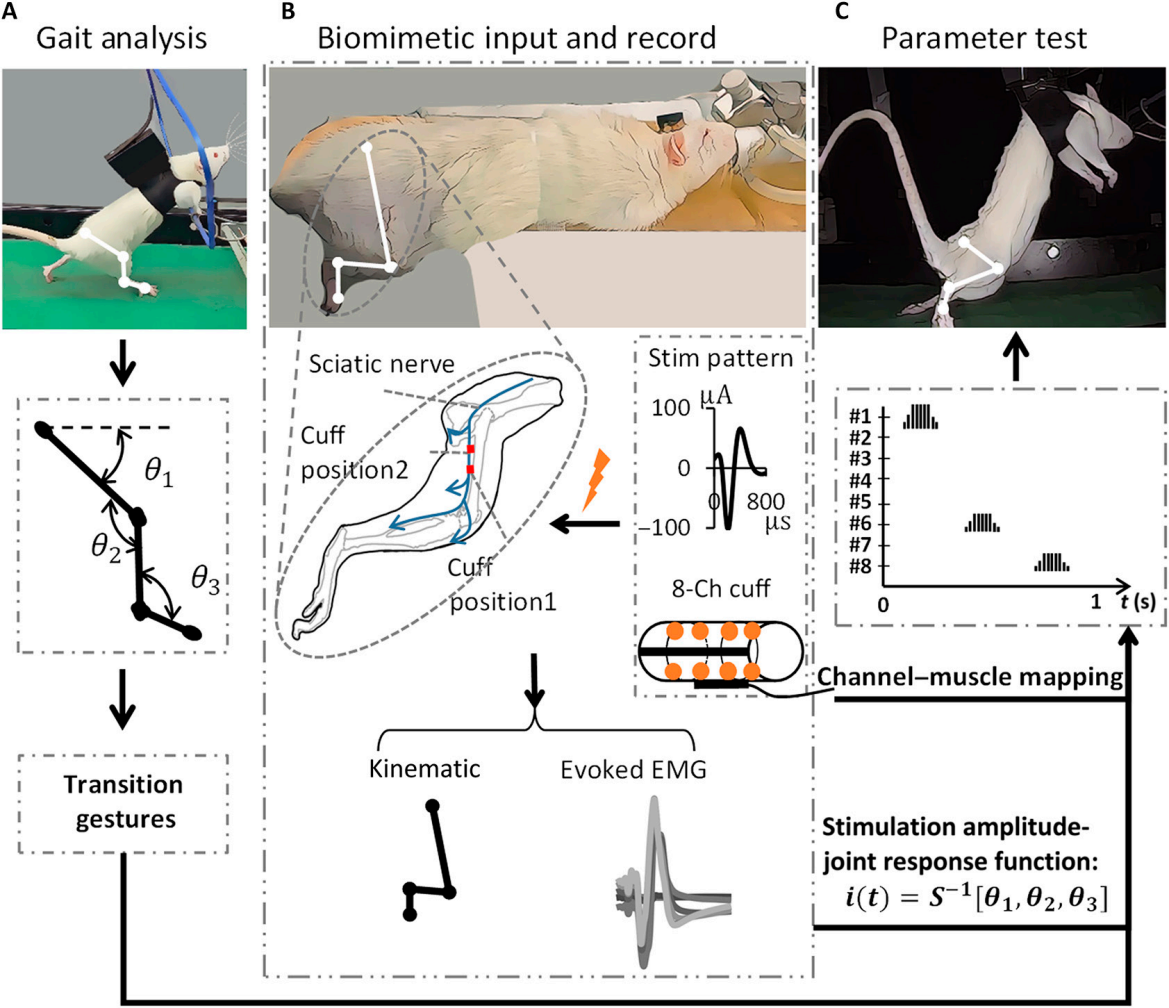
Experimental setup and design. (A) Rat hindlimb motion capture for gait analysis. Four markers were attached to the hip joint, knee joint, ankle joint, and foot, respectively, to record the hindlimb’s trajectory. The hindlimb model has been simplified into a stick diagram for joint kinematics analysis: *θ*_1_, the hip joint; *θ*_2_, the knee joint; *θ*_3_, the ankle joint. (B) The biomimetic stimulation of the sciatic nerve and the parameters’ settings. The SSP was 100 μA in amplitude and 800 μs in width. The 8-channel cuff (8-Ch cuff) was implanted in position 1 or position 2. The kinematic and evoked EMG data were recorded for parameter optimization. (C) Parameter testing was carried out on the anesthetized rats on the treadmill. The hindlimb motion was recorded for evaluation. The relationship between stimulation amplitude and joint response was described by a formula, where *i*(*t*) represent the stimulation electric current, *θ* represent 3 joint angles, and *S*^−1^ represent the inverse operation of sigmoid function.

During surgery, the rats were anesthetized with isoflurane in oxygen-enriched air (1.5% to 2.5%). Small incisions were made to expose the skulls and hindlimb muscles. The vastus lateralis (VL) and biceps femoris (BF) were separated, and the sciatic nerve was exposed. The tissue was removed from its surface. A 3-mm-long cuff electrode of 1 mm in diameter (2 × 4 contacts; Kedou Brain Computer Technology Co. Ltd., Suzhou, China) was used for sciatic nerve stimulation. Its contacts (0.1 mm in diameter; uniformly distributed on the circle) were made with Pt and embedded with silicone. The cuff electrode was fastened near the small branch of the sciatic nerve, which innervated BF that would be found at the middle segment of sciatic nerve and near the middle of femur (the sketch map was illustrated in Fig. 1B). Four cuff electrodes were implanted in position 1, and the other 2 electrodes were set in position 2, which was approximately 10 mm from position 1. The ground wire of the cuff was placed subcutaneously in the rat’s back. Bipolar electrodes (Teflon-coated stainless-steel wires, Kedou Brain Computer Technology Co. Ltd., Suzhou, China) were implanted into the 4 left hindlimb muscles: tibialis anterior (TA), gastrocnemius muscle (GM), BF, and VL. These electrodes recorded the evoked EMG signals for evaluating the effect of stimulation. The wires were routed subcutaneously into the Samtec connectors fixed to the rat’s skull with dental cement.

### Experiment design

For gait analysis, the rats were trained to bipedally walk on a homemade treadmill for the first 2 weeks (Fig. 1A, 5 to 6 d/week, 30 min/d) [40]. After the rats completed the training, their hindlimb motion was recorded using the motion capture system. Once the gait data were completely recorded, the cuff and EMG electrodes were implanted into the rats, and the rats were allowed to rest for 2 weeks. Next, the rats were anesthetized and fixed on a custom platform that allowed the right hindlimb to hang to perform sciatic nerve stimulation (Fig. 1B). The stimulator delivered the current in the established order while adhering to the channel, and the hindlimb motion and evoked EMG signals were recorded by the acquisition system. During the stimulation, each parameter was repeated 20 times. Finally, the anesthetized rat was hung on the treadmill, and the appropriate stimulus amplitude and sequence parameters were selected for hindlimb modulation (Fig. 1C).

The biomimetic concept inspired us to draw creativity from the natural world [41]. We extracted an action potential signal from the recorded sciatic nerve signals during rats bipedally walking and scaled it in an amplitude of −100 μA (Fig. 1, stim pattern). We define this scaled action potential as a standard stimulus pattern (SSP). By amplitude modulation and frequency modulation, a stimulus sequence, called biomimetic input, was used for sciatic nerve stimulation. In a previous study, the charge injection of the spike waveform was lower than that of the square waveform at the same activation threshold [42]. Figure 1B presents the waveform of the pattern. The amplitude was set at −100 μA, the pulse width was 800 μs, and the amplitude used in the experiment ranged from 0.2 to 1 × SSP. The stimulus frequency was set at 5 and 50 Hz for the parameter test and motion modulation, respectively.

### Data recording and analysis

Markers were attached to the hips, knees, ankles, and foot of the rats’ right hindlimbs (Fig. 1A). An AVT camera (Allied Vision Technologies, Stadtroda, Germany) was used for motion capture, with its sampling rate being 80 fps. The resolution was set at 640 × 480 dpi. Using the Plexon system, the EMG signal was recorded at a 40-kHz sampling rate.

In our experiment, the rats were anesthetized, so as to evoke EMG signals through stimulation. These signals were read using Plexon software development kit and filtered through a bandpass filter (300 to 3,000 Hz). The dc offset was removed. The experiment included 144 groups with channel–amplitude–frequency combinations (8 cuff channels, 9 stimulus amplitudes, and 2 stimulus frequency parameters). The mean peak-to-peak value of 20 trials was used to assess the effects of different stimulation parameters.

### Selectivity index

The peak-to-peak values (Vpp) of the evoked EMG were normalized to the maximum value of compound muscular action potential to represent the response of stimulation (*r*, ranged from 0% to 100%). For each stimulation channel (Ch) and stimulation intensity (*I*), the selectivity index (*SI*) of each muscle (*m*) was calculated as follows:

$$\mathrm{SI}=\frac{r_{\mathrm{Ch},m}\left(I\right)}{\sum_{j=0}^{4}r_{\mathrm{Ch},j}\left(I\right)}$$ (1)

### Statistics analysis

Statistical analysis was performed using SPSS software (version 27.0, IBM Corp., Armonk, NY, USA). Two-way analysis of variance (ANOVA) was performed to analyze the effects between the normal and stimulation states. The interaction between the 2 factors was first determined using a simple effect test. Then, if the statistical significance of 2-way interaction was observed between them, the Tukey method was used for post hoc testing to compare the groups. The significance level for all tests was set at *P* < 0.05. GraphPad Prism version 9.0 (GraphPad Software, La Jolla, CA, USA) was used for graph construction.

## Results

### Motion gestures produced by nerve electrical stimulation

**Fig. 2.**
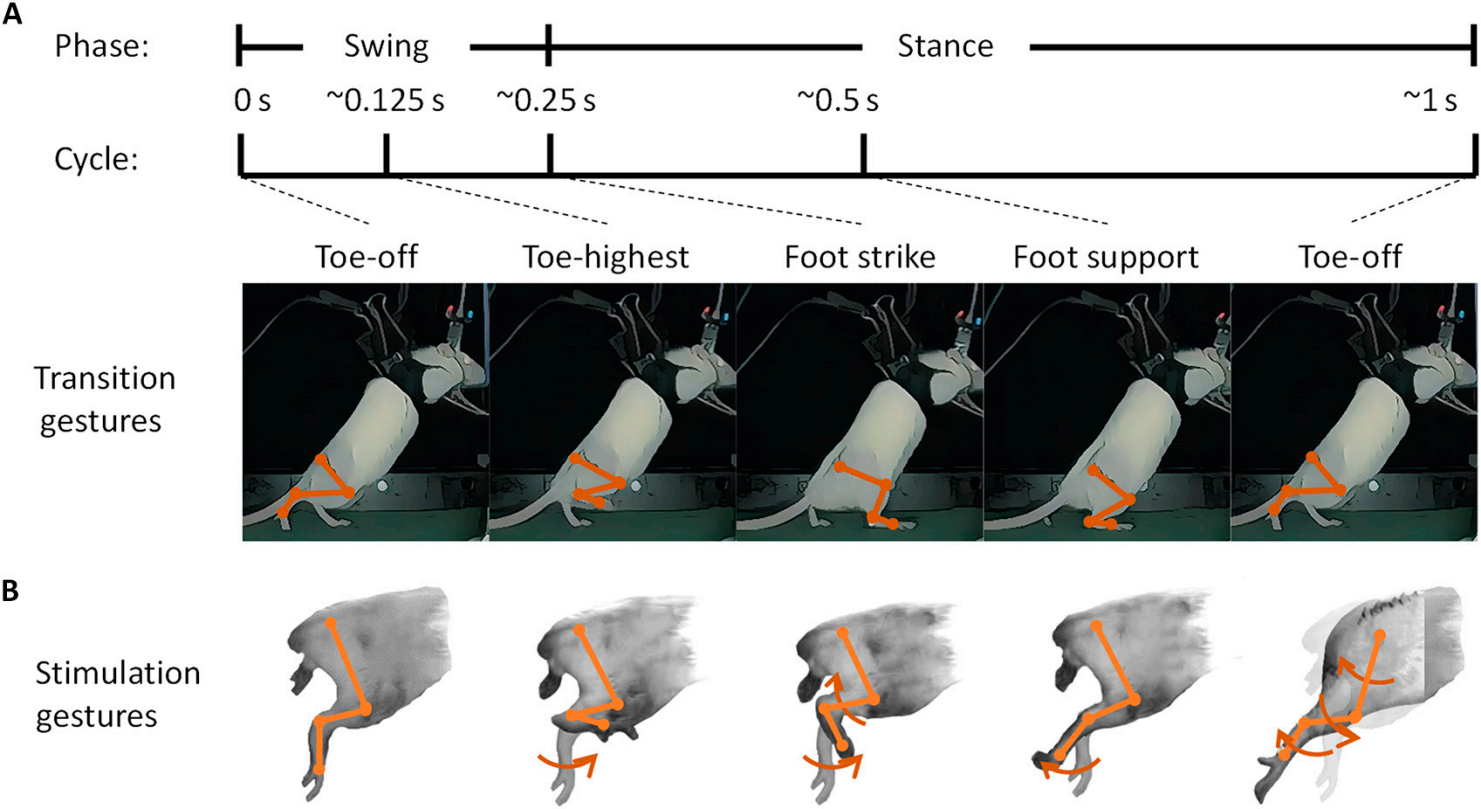
Hindlimb gestures recorded by motion capture system under self-bipedal walking and stimulated by electrical stimulation system. (A) Four typical movement postures of rats during the rats stepping on the treadmill. (B) The stimulation gestures of hindlimb: foot dorsiflexion; foot plantarflexion; knee flexion and foot dorsiflexion; and hip, knee, and ankle extension.

Initially, to select the postures, we recorded 6 rats that were suspended on the treadmill and were bipedally walking on it during the whole cycle. By referring to the Rancho Los Amigos classification method, the 4 transition actions of the unilateral lower limb were extracted: Toe-off, Toe-highest, Foot strike, and Foot support (Fig. 2A). Here, Toe-off represents the movement that the toe left the ground and flexors were strengthened; Toe-highest represents the movement that the toe reached the highest position; Foot strike represents the movement that the foot touched the ground; Foot support represents the movement that the unilateral lower limb supported the body. These 4 postures were established as the target in sciatic nerve electrical stimulation to judge whether the stimulation could effectively activate lower limb joint activity.

In various recruited hindlimb movements, the typical stimulation gestures were found in the video capture. Figure 2B presents the results of hindlimb motion modulation in rat #21 through electrical stimulation in sequence under 0.8 SSP. The hip, knee, and ankle joints were subjected to different stimulation levels. The TA muscle group was effectively activated through channels 1, 2, 5, and 6, thereby causing foot dorsiflexion, wherein the angle reached 13.2° ± 0.49° (mean ± SEM). The GM muscle group was activated through channel 1, thereby causing foot plantarflexion, wherein the angle was 132.5° ± 1.44° (mean ± SEM). The use of channels 1, 2, 5, and 6 with high stimulus intensity resulted in combined motion, including foot dorsiflexion and knee flexion. Similarly, hindlimb extensors stretched when channel 8 was used.

Then, the stimulating effects of single-cathode nerve electrical stimulation applied to the proximal and distal ends of the sciatic nerve, respectively, were validated. With some electrode configurations, the isolated movements principally included foot flexion and dorsiflexion at a low stimulus amplitude. The foot movements were obtained for all 6 subjects. Table 1 presents the effective channels used to achieve foot modulation in each rat. However, with an increase in the stimulus intensity, the joint movement angle increased, and the compound gestures of the hindlimb appeared with a high possibility.

**Table 1.** Hindlimb reflexes following stimulation using 2 implant positions and 8 channels

		Foot		Knee		Hip	
Cuff position	Rat	Flexion	Extension	Flexion	Extension	Flexion	Extension
1	#11	1, 2, 5, 6	3, 7	**1, 2, 5, 6**	–	–	–
	#12	1, 4, 5, 8	2, 3, 6, 7	**1, 4, 5, 8**	–	–	–
	#13	1, 4, 5	2, 3, 7	8	–	–	–
	#14	1, 4, 5, 8	2, 3, 6, 7	**1, 4, 5, 8**	–	–	–
2	#21	1, 2, 5, 6	3, 4, 7, 8	**1, 2, 5, 6**	8	–	8
	#22	1, 4	2, 3, 5, 6, 7, 8	**1, 4**	5,6	–	5,6

The bolded numbers, such as **1**, illustrated the motion that would be activated with high stimulus intensity through channel 1.

The combined movements usually appeared when the knee flexed or the hip extended under electrical stimulation in both the implantation positions. The results illustrated that we could still activate the composite movements of lower limb joints, regardless of electrode implantation at the proximal or distal end of the sciatic nerve, even under low-intensity neural electrical stimulation. Specifically, stimulation applied at the proximal sciatic nerve segment effectively activated the muscles on the posterior side of the thigh, thereby manifesting as extension muscle force for extending the lower limb. Neural electrical stimulation applied at the middle sciatic nerve segment primarily led to the combined movement of knee and ankle joints.

The coordinated flexion of the knee–ankle joints allowed the foot to rapidly leave the ground (in rat #13, channel 8). Furthermore, as the stimulation intensity increased gradually, a transition gesture was observed from the ankle joint flexion to the knee–ankle joint flexion. The bold numbers in Table 1 illustrated that single movement activated in low stimulus amplitude and combined movements recruited in high stimulation intensity.

### The sigmoid characteristic curve of the evoked joint movement and EMG response

To corroborate the relationship between the neural electrical stimulation intensity and lower limb joint movement, the data of various joint movements under the same parameter stimulation were recorded for 20 trials. On the basis of the statistically analyzed experimental results, a sigmoidal relationship was observed between the extraneural electrical stimulation intensity and joint movement angle. The CurveFitter tool was used to calculate the sigmoidal function parameters and the similarity. Table 2 presents the specific parameters for stimulation channels.

**Table 2.** The parameters of sigmoid function in joints responses

Parameters		*a*	*b*	*c*	*d*	*R* ^2^
Ankle	Ch2	1.7877	13.9344	62.2197	−83.4064	0.998932
	Ch4	−15.7679	13.6311	61.5864	−100.727	0.992258
	Ch6	1.12293	15.4462	57.2092	−85.6609	0.99977
Knee	Ch2	0.023459	6.94024	68.555	−31.7297	0.964527
	Ch4	36884.2	1.5948	0.555037	−33.9924	0.969152
	Ch6	4.14224	4.56795	75.8536	−47.1865	0.980712
	Ch8	19.5587	2.12066	111.192	−74.1934	0.950457

**Fig. 3.**
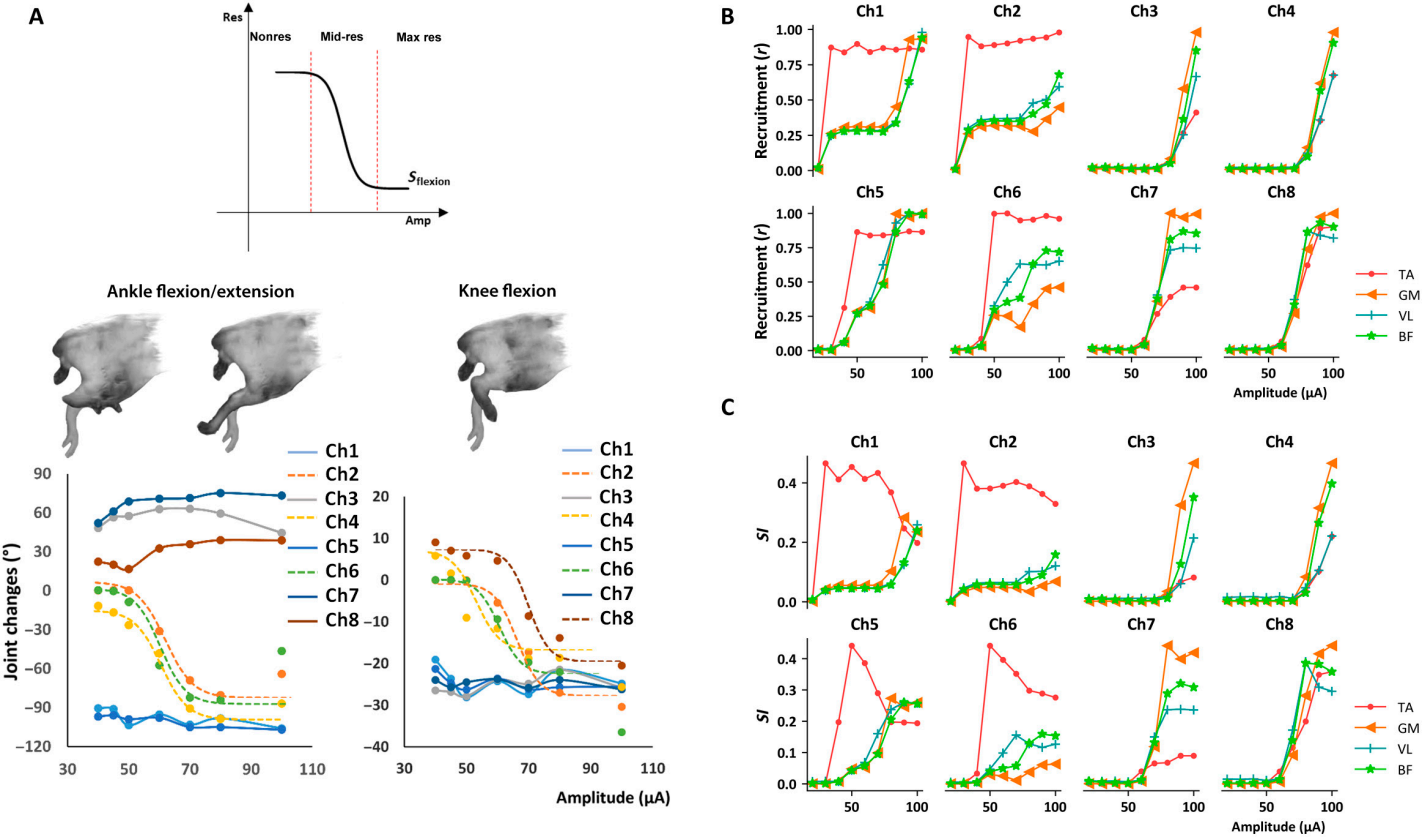
Joint angle changes and EMG signals that were evoked with different amplitude and channel settings. (A) The standard sigmoid function curves and changes in the hindlimb joint when the stimulus amplitude increased from 0.4 to 0.8 SSP. (B and C) The recruitments and selectivity indexes of muscles at various amplitudes of stimulus.

On the basis of the response of joints after stimulation and sigmoidal function characteristics, the stimulation effects were divided into 3 stages: nonresponse, mid-response, and max response (Fig. 3A). Within the selected range of stimulation intensities, some channels (channels 1, 3, 5, 7, and 8) caused changes in joint angles, which reached the limits of flexion or extension after electrical stimulation application (Fig. 3A). By contrast, for other channels (channels 2, 4, and 6), the response of each joint exhibited the 3 periods with an increase in the stimulation intensity. The results of these 3 channels (channels 2, 4, and 6) were statistically analyzed, and their curve fitting coefficients were 0.9989, 0.9922, and 0.9998, respectively. This indicated a good fit to the pattern of changes in joint angles with an increase in the stimulation intensity. Similar curve characteristics were observed during the change process of the knee joint angle, with fitting coefficients of 0.9645, 0.9692, 0.9807, and 0.9506, reflecting the pattern of changes in joint angles with an increase in the stimulation intensity.

### Performances of combination parameter tests on the treadmill

**Fig. 4.**
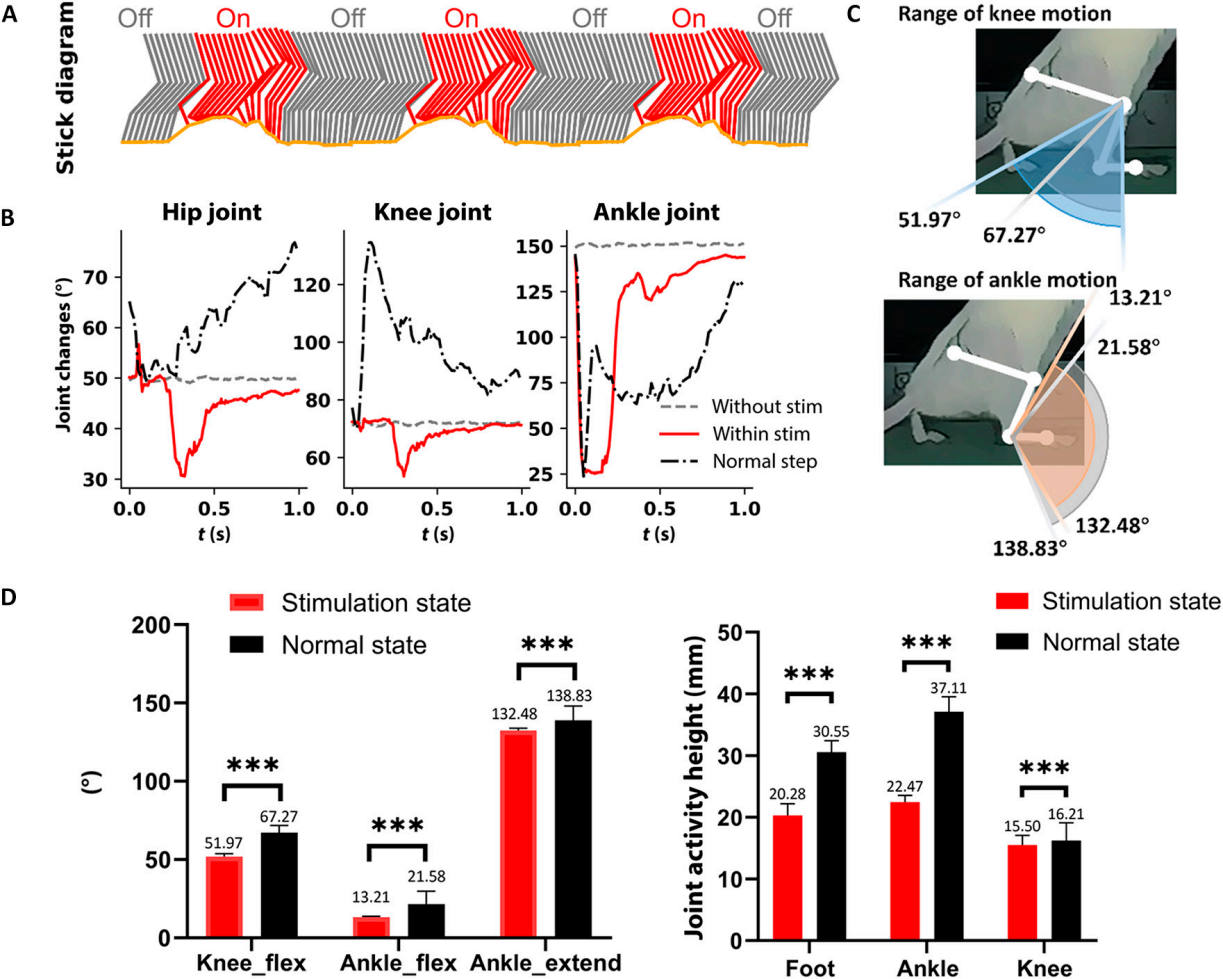
Hindlimb modulation via sciatic nerve stimulation on the treadmill. (A) A stick diagram of hindlimb motion. (B) The differences in hip/knee/ankle joint angle changes among tests of normal hindlimb step (black), stimulation off (gray), and stimulation on (red). (C) The range of motion in knee and ankle. (D) The motion ranges and height changes of rat joints between self-walking state and stimulation state. ***P < 0.001.

We here compared among the normal stepping state and anesthetized states with and without stimulation. For this comparison, the anesthetized rats were suspended on a treadmill, and the treadmill speed was set up to 3 cm/s before the parameter test. Initially, the rats generated a drag gait. The state of hindlimb was used for the comparison (Fig. 4A, gray line in stick diagram). The angles of the hip, knee, and ankle joints were 50.13°, 72.35°, and 151.48°, respectively. On the basis of the guideline of channel–joint mapping relationship (Table 1), we combined the channels that were effective in modulating the hindlimb joints. The red lines in the stick diagram are the hindlimb-modulated states following sciatic nerve stimulation (Fig. 4A). The changes of the 3 joint angles were 25.68° ± 1.43°, 17.61° ± 1.95°, and 124.20° ± 2.68° (means ± SEM) in the hip, knee, and ankle, respectively, with the activation of the electrical stimulation (Fig. 4B).

Moreover, the difference between the curves of the normal stepping state and the stimulation state illustrated the effect of sciatic nerve stimulation. During the first half second, the stimulation induced a flex in the control of the ankle joint, thereby causing the foot to leave the ground. This lasted for approximately 0.2 to 0.3 s. Then, the stimulation activated the combined movement to straighten the hindlimb, so that it supports the trunk until the end. During a stimulation cycle, the foot flexion angle changed from 138.84° to 13.21°, the knee extension angle changed from 51.97° to 71.07°, and the hip extension angle changed from 30.54° to 47.56° (Fig. 4C and D). The overlap ratio in ankle motion was up to 94.58%.

## Discussion

In this study, the classic multichannel cuff electrodes were used to perform extraneural electrical stimulation of the sciatic nerve of 6 rats, thereby precisely controlling multiple effective lower limb movements of the rats to regulate these movements in the unconscious state. Through single-point cathodal stimulation, we could accurately control the knee and ankle joint actions and generate compound knee–ankle and hip–knee–ankle joint movements based on different stimulation intensities. By analyzing the joint motion data, the characteristic sigmoid function relationship between the stimulation intensity and joint changes was verified, thereby allowing us to reverse engineer the function of intensity changes and achieve the goal of predefined trajectory control under specified joint movement conditions. In parameter testing, the effects of stimulation parameters and the combination of stimulation intensities and durations, which were selected on the basis of the channel–joint mapping and the sigmoid function, were estimated. According to the results, sciatic nerve electrical stimulation can accurately modulate hindlimb joint activity in anesthetized rats. When the stimulation intensity is adjusted, coordinated contraction effects can be achieved at hip, knee, and ankle joints, thereby effectively controlling the joint movements.

The Rancho Los Amigos gait analysis methods [43], proposed by National Rehabilitation Center in California, categorizes the whole gait cycle into 8 periods based on the state of contact between the feet and ground, which is beneficial for diagnosing lower limb diseases. However, in patients with the loss of sensory and motor function of lower extremity, we used nerve electrical stimulation to regulate the lower extremity muscle movement to assist them in completing specific actions. In our experiment, we focused more on the limit position of joint movements, so as to adjust the stimulus parameters for modulating unilateral lower limb movement. We selected 4 typical motion gestures from the gait cycle, namely, foot strike, foot support, toe-off, and toe-highest, for calculating joint modulating ranges. Furthermore, in rats subjected to bipedal walking, the charge of stimulation from one compound movement to another was lower than each single joint modulation, whereas the efficiency of compound movement modulation was higher than that of continuous single motion control.

We used multicontact cuff electrodes to verify that application of single-cathode neural electrical stimulation can help in successfully controlling muscles, such as the TA, GM, VL, and quadriceps, exclusively by regulating ankle and knee joints. The stimulation intensity used in our experiment was only slightly different from that within the nerve bundle, with the charge threshold (10 to 20 nC in ours) being only 2 to 3 times that of the nerve bundle [44]. Meanwhile, single-point neural electrical stimulation had lower intensity requirements than multipoint combined stimulation [45]. Consequently, these stimulation parameters guarantee the activation of lower limb muscles within a safe stimulation range [46]. Furthermore, in previous studies, knee joint muscles were not effectively controlled solely through sciatic nerve electrical stimulation. Applying electrical stimulation on or within the muscles, such as the quadriceps, to regulate their function was a common approach. By adjusting the positions along the sciatic nerve bundle, we achieved coordinated flexion of hip, knee, and ankle joints. This unlocks new possibilities for the further functional recovery of lower limb movement through sciatic nerve control.

In addition, a mapping between stimulation channels and joint movements was proposed here. We verified the sigmoid-shaped characteristic curve of joint angle variation with a stimulation current. In a previous neural electrical stimulation model, Bucksot et al. [47] established that the stimulation site, electrode size, and stimulation intensity affect nerve bundle activation. They observed that the nerve bundle activation rate presented an S-shaped characteristic curve under the sole condition of varying stimulation intensities. This indirectly provided a foundation for the relationship between the stimulation intensity and joint flexion observed in our experiment. However, a small number of points were not fit to the curve. The most nerve bundles were recruited by the high stimulation intensity across the section to activate the joints simultaneously, which possibly caused the angle changes smaller than the predicted value. As observed through the recruitment and selectivity index results, the *r* and selectivity index of the muscles became lower at a high intensity than at a low intensity.

On analyzing the statistical results, we found that the electrical-stimulation-regulated angles of hip flexion, ankle flexion, and ankle extension were 77.24%, 61.21%, and 95.43% of those in the bipedal walking state of the lower limb, respectively (Fig. 4C). Moreover, the heights of the foot, ankle, and knee modulation were 66.38%, 60.55%, and 95.56% (Fig. 4D), respectively, which were significantly lower than the percentages in the heights. Thus, we indirectly verified that the control of sciatic nerve electrical stimulation over actions such as hip flexion and knee extension is weaker. Therefore, extraneural electrical stimulation of the sciatic nerve can effectively control posterior thigh muscles and joints, such as the foot and ankle, but not the hip flexion and knee extension.

### Limitation and future work

Successful results were obtained in hindlimb motion modulation because the sciatic nerve has a large diameter and exhibited clear functional division and the extrafascicular electrode was a good working state. However, some limitations of our experiment remain. On one hand, sciatic nerve electrical stimulation has limited control over the hip joint. To perfectly control the lower limb movement, neural stimulation from other areas must be combined. On the other hand, single-cathode nerve electrical stimulation offers good advantages in regulating compound movements but exhibits an average performance in precise control of single joints. Finally, parameter optimization based on sciatic nerve regulation is complex. Developing an optimization algorithm would therefore be more effective in facilitating safety exploration.

### Conclusion

We here proposed a method for facilitating hindlimb motor regulation through extraneural electrical stimulation of the sciatic nerve. First, the method verified that proximal sciatic nerve electrical stimulation can effectively activate posterior thigh muscles and distal nerve electrical stimulation can precisely control knee and ankle joint movement. Then, the method mapped the relationships between nerve bundle segments and joint movements and allows the activation of specific parts of nerve fiber bundles, so as to manipulate lower limb muscles. The kinematics and EMG results were then used to determine the appropriate stimulation intensity, so as to reduce the electrical-stimulation-induced potential neural tissue damage. Moreover, the sigmoid function between the stimulus intensity and joint movements allowed inverse operation under the planned motion trajectory. The proposed neuromodulation method could help reduce the difficulty of surgery and the electrical nerve stimulation intensity, thus offering a new approach for regulating and rehabilitating hindlimb dysfunction.

## Data Availability

The data presented in this study are available on request from the corresponding author. The data are not publicly available due to the need for further analysis.

## References

[1] McHugh C, Taylor C, Mockler D, Fleming N. Epidural spinal cord stimulation for motor recovery in spinal cord injury: A systematic review. NeuroRehabilitation. 2021;49(1):1–22. 33967072 10.3233/NRE-210093

[2] Megía García A, Serrano-Muñoz D, Taylor J, Avendaño-Coy J, Gómez-Soriano J. Transcutaneous spinal cord stimulation and motor rehabilitation in spinal cord injury: A systematic review. Neurorehabil Neural Repair. 2020;34(1):3–12. 31858871 10.1177/1545968319893298

[3] Rejc E, Angeli CA, Bryant N, Harkema SJ. Effects of stand and step training with epidural stimulation on motor function for standing in chronic complete paraplegics. J Neurotrauma. 2017;34(9):1787–1802. 27566051 10.1089/neu.2016.4516 PMC5421606

[4] Rowald A, Komi S, Demesmaeker R, Baaklini E, Hernandez-Charpak SD, Paoles E, Montanaro H, Cassara A, Becce F, Lloyd B, et al. Activity-dependent spinal cord neuromodulation rapidly restores trunk and leg motor functions after complete paralysis. Nat Med. 2022;28(2): 260–271. 35132264 10.1038/s41591-021-01663-5

[5] Malešević J, Konstantinović L, Bijelić G, Malešević N. Smart protocols for physical therapy of foot drop based on functional electrical stimulation: A case study. Healthcare. 2021;9(5):502. 33925814 10.3390/healthcare9050502 PMC8146368

[6] Kubota S, Kadone H, Shimizu Y, Koda M, Noguchi H, Takahashi H, Watanabe H, Hada Y, Sankai Y, Yamazaki M. Development of a new ankle joint hybrid assistive limb. Medicina. 2022;58(3):395. 35334571 10.3390/medicina58030395 PMC8955947

[7] Müller P, del Ama AJ, Moreno JC, Schauer T. Adaptive multichannel FES neuroprosthesis with learning control and automatic gait assessment. J NeuroEngineering Rehabil. 2020;17(1):36. 10.1186/s12984-020-0640-7 PMC7048130 32111245

[8] Berenpas F, Weerdesteyn V, Geurts AC, van Alfen N. Long-term use of implanted peroneal functional electrical stimulation for stroke-affected gait: The effects on muscle and motor nerve. J NeuroEngineering Rehabil. 2019;16(1):86. 10.1186/s12984-019-0556-2 PMC6621964 31292003

[9] Bethoux F, Rogers HL, Nolan KJ, Abrams GM, Annaswamy T, Brandstater M, Browne B, Burnfield JM, Feng W, Freed MJ, et al. Long-term follow-up to a randomized controlled trial comparing peroneal nerve functional electrical stimulation to an ankle foot orthosis for patients with chronic stroke. Neurorehabil Neural Repair. 2015;29(10):911–922. 25653225 10.1177/1545968315570325

[10] Fisher LE, Miller ME, Bailey SN, Davis Jr JA, Anderson JS, Rhode L, Tyler DJ, Triolo RJ. Standing after spinal cord injury with four-contact nerve-cuff electrodes for quadriceps stimulation. IEEE Trans Neural Syst Rehabil Eng. 2008;16(5):473–478. 18990650 10.1109/TNSRE.2008.2003390 PMC2936226

[11] Hausmann J, Sweeney-Reed CM, Sobieray U, Matzke M, Heinze HJ, Voges J, Buentjen L. Functional electrical stimulation through direct 4-channel nerve stimulation to improve gait in multiple sclerosis: A feasibility study. J NeuroEngineering Rehabil. 2015;12:100. 10.1186/s12984-015-0096-3 PMC4650371 26577467

[12] Losanno E, Badi M, Wurth S, Borgognon S, Courtine G, Capogrosso M, Rouiller EM, Micera S. Bayesian optimization of peripheral intraneural stimulation protocols to evoke distal limb movements. J Neural Eng. 2021;18(6):6066046. 10.1088/1741-2552/ac3f6c 34874320

[13] Rijnbeek EH, Eleveld N, Olthuis W. Update on peripheral nerve electrodes for closed-loop neuroprosthetics. Front Neurosci. 2018;12:350. 29910705 10.3389/fnins.2018.00350 PMC5992394

[14] Qu J, Mao B, Li Z, Xu Y, Zhou K, Cao X, Fan Q, Xu M, Liang B, Liu H, et al. Recent progress in advanced tactile sensing technologies for soft grippers. Adv Funct Mater. 2023;33(41):2306249.

[15] Ryan JL, Eng E, Fehlings DL, Wright FV, Levac DE, Beal DS. Motor evoked potential amplitude in motor behavior-based transcranial direct current stimulation studies: A systematic review. J Mot Behav. 2023;55(3):313–329. 36919517 10.1080/00222895.2023.2184320

[16] Czarnecki P, Huber J, Szukała A, Górecki M, Romanowski L. The usefulness of motor potentials evoked transvertebrally at lumbar levels for the evaluation of peroneal nerve regeneration after experimental repair in rats. J Pers Med. 2023;13(3):438. 36983619 10.3390/jpm13030438 PMC10054717

[17] Gerasimenko YP, Ichiyama RM, Lavrov IA, Courtine G, Cai L, Zhong H, Roy RR, Edgerton VR. Epidural spinal cord stimulation plus quipazine administration enable stepping in complete spinal adult rats. J Neurophysiol. 2007;98(5):2525–2536. 17855582 10.1152/jn.00836.2007

[18] Lavrov I, Courtine G, Dy CJ, van den Brand R, Fong AJ, Gerasimenko Y, Zhong H, Roy RR, Edgerton VR. Facilitation of stepping with epidural stimulation in spinal rats: Role of sensory input. J Neurosci. 2008;28(31):7774–7780. 18667609 10.1523/JNEUROSCI.1069-08.2008 PMC2897701

[19] van den Brand R, Heutschi J, Barraud Q, DiGiovanna J, Bartholdi K, Huerlimann M, Friedli L, Vollenweider I, Moraud EM, Duis S, et al. Restoring voluntary control of locomotion after paralyzing spinal cord injury. Science. 2012;336(6085):1182–1185. 22654062 10.1126/science.1217416

[20] Vromans M, Faghri P. Electrical stimulation frequency and skeletal muscle characteristics: Effects on force and fatigue. Eur J Transl Myol. 2017;27(4): Article 6816. 29299218 10.4081/ejtm.2017.6816 PMC5745385

[21] Gruner JA, Mason CP. Nonlinear muscle recruitment during intramuscular and nerve stimulation. J Rehabil Res Dev. 1989;26(2):1–16. 2724148

[22] Ragnarsson KT. Functional electrical stimulation after spinal cord injury: Current use, therapeutic effects and future directions. Spinal Cord. 2008;46(4):255–274. 17846639 10.1038/sj.sc.3102091

[23] Schiefer MA, Freeberg M, Pinault GJC, Anderson J, Hoyen H, Tyler DJ, Triolo RJ. Selective activation of the human tibial and common peroneal nerves with a flat interface nerve electrode. J Neural Eng. 2013;10(5):056006. 23918148 10.1088/1741-2560/10/5/056006 PMC3809099

[24] Schiefer MA, Polasek KH, Triolo RJ, Pinault GCJ, Tyler DJ. Selective stimulation of the human femoral nerve with a flat interface nerve electrode. J Neural Eng. 2010;7(2):026006. 10.1088/1741-2560/7/2/026006 PMC2915830 20208125

[25] Burridge J, Haugland M, Larsen B, Pickering RM, Svaneborg N, Iversen HK, Christensen PB, Haase J, Brennum J, Sinkjaer T. Phase II trial to evaluate the ActiGait implanted drop-foot stimulator in established hemiplegia. J Rehabil Med. 2007;39(39):212–218. 17468789 10.2340/16501977-0039

[26] Chen G, Ma L, Song R, Li L, Wang X, Tong K. Speed-adaptive control of functional electrical stimulation for dropfoot correction. J NeuroEngineering Rehabil. 2018;15(1):98. 10.1186/s12984-018-0448-x PMC6220509 30400918

[27] Ernst J, Grundey J, Hewitt M, von Lewinski F, Kaus J, Schmalz T, Rohde V, Liebetanz D. Towards physiological ankle movements with the ActiGait implantable drop foot stimulator in chronic stroke. Restor Neurol Neurosci. 2013;31(5):557–569. 23756541 10.3233/RNN-120283

[28] Liberson WT, Holmquest HJ, Scot D, Dow M. Functional electrotherapy: Stimulation of the peroneal nerve synchronized with the swing phase of the gait of hemiplegic patients. Arch Phys Med Rehabil. 1961;42:101–105. 13761879

[29] Song K-I, Park SE, Hwang D, Youn I. Compact neural interface using a single multichannel cuff electrode for a functional neuromuscular stimulation system. Ann Biomed Eng. 2019;47(3):754–766. 30560306 10.1007/s10439-018-02181-1

[30] Thota AK, Kuntaegowdanahalli S, Starosciak AK, Abbas JJ, Orbay J, Horch KW, Jung R. A system and method to interface with multiple groups of axons in several fascicles of peripheral nerves. J Neurosci Methods. 2015;244:78–84. 25092497 10.1016/j.jneumeth.2014.07.020 PMC4312748

[31] Malagodi MS, Horch KW, Schoenberg AA. An intrafascicular electrode for recording of action potentials in peripheral nerves. Ann Biomed Eng. 1989;17(4):397–410. 2774314 10.1007/BF02368058

[32] Branner A, Stein RB, Normann RA. Selective stimulation of cat sciatic nerve using an array of varying-length microelectrodes. J Neurophysiol. 2001;85(4):1585–1594. 11287482 10.1152/jn.2001.85.4.1585

[33] Veraart C, Grill WM, Mortimer JT. Selective control of muscle activation with a multipolar nerve cuff electrode. IEEE Trans Biomed Eng. 1993;40(7):640–653. 8244425 10.1109/10.237694

[34] Brill NA, Tyler DJ. Quantification of human upper extremity nerves and fascicular anatomy. Muscle Nerve. 2017;56(3): 463–471. 28006854 10.1002/mus.25534 PMC5712902

[35] Brill NA, Naufel SN, Polasek K, Ethier C, Cheesborough J, Agnew S, Miller LE, Tyler DJ. Evaluation of high-density, multi-contact nerve cuffs for activation of grasp muscles in monkeys. J Neural Eng. 2018;15(3):036003. 28825407 10.1088/1741-2552/aa8735 PMC5910281

[36] Dali M, Rossel O, Guiraud D. Fast simulation and optimization tool to explore selective neural stimulation. Eur J Transl Myol. 2016;26(3):6060. 27990231 10.4081/ejtm.2016.6060 PMC5128964

[37] Dali M, Guiraud D. Modeling peripheral nerve stimulation. In: Thakor NV, editor. *Handbook of neuroengineering*. Singapore: Springer Nature Singapore; 2023. p. 927–973.

[38] Tigra W, Dali M, William L, Fattal C, Gélis A, Divoux JL, Coulet B, Teissier J, Guiraud D, Azevedo Coste C. Selective neural electrical stimulation restores hand and forearm movements in individuals with complete tetraplegia. J Neuroeng Rehabil. 2020;17(1):66. 32429963 10.1186/s12984-020-00676-4 PMC7236876

[39] Coste CA, William L, Fonseca L, Hiairrassary A, Andreu D, Geffrier A, Teissier J, Fattal C, Guiraud D. Activating effective functional hand movements in individuals with complete tetraplegia through neural stimulation. Sci Rep. 12(1):16189. 10.1038/s41598-022-19906-x PMC9537317 36202865

[40] Lang Y, Tang R, Liu Y, Xi P, Liu H, Quan Z, Song D, Lv X, Huang Q, He J. Multisite simultaneous neural recording of motor pathway in free-moving rats. Biosensors. 2021;11(12):503. 34940260 10.3390/bios11120503 PMC8699182

[41] Bing Z, Rohregger A, Walter F, Huang Y, Lucas P, Morin FO, Huang K, Knoll A. Lateral flexion of a compliant spine improves motor performance in a bioinspired mouse robot. Sci Robot. 2023;8(85):eadg7165. 38055804 10.1126/scirobotics.adg7165

[42] Dali M, Picq C, Rossel O, Maciejasz C-HM, Guiraud D. Comparison of the efficiency of chopped and non-rectangular electrical stimulus waveforms in activating small vagus nerve fibers. J Neurosci Methods. 2019;320:1–8. 30826387 10.1016/j.jneumeth.2019.02.017

[43] Whittle MW. *Gait analysis: An introduction - 3rd edition*. Oxford (UK): Butterworth-Heinemann; 2003.

[44] Badia J, Boretius T, Andreu D, Azevedo-Coste C, Stieglitz T, Navarro X. Comparative analysis of transverse intrafascicular multichannel, longitudinal intrafascicular and multipolar cuff electrodes for the selective stimulation of nerve fascicles. J Neural Eng. 2011;8(3):036023. 21558601 10.1088/1741-2560/8/3/036023

[45] Tarler MD, Mortimer JT. Comparison of joint torque evoked with monopolar and tripolar-cuff electrodes. IEEE Trans Neural Syst Rehabil Eng. 2003;11(3):227–235. 14518785 10.1109/TNSRE.2003.816867

[46] Elyahoodayan S, Larson C, Cobo AM, Meng E, Song D. Acute in vivo testing of a polymer cuff electrode with integrated microfluidic channels for stimulation, recording, and drug delivery on rat sciatic nerve. J Neurosci Methods. 2020;336: Article 108634. 32068010 10.1016/j.jneumeth.2020.108634

[47] Bucksot JE, Chandler CR, Intharuck NM, Rennaker RL, Kilgard MP, Hays SA. Validation of a parameterized, open-source model of nerve stimulation. J Neural Eng. 2021;18(4): Article ac1983. 10.1088/1741-2552/ac1983 PMC9533340 34330105

